# The Octopus Mastopexy: A True “Implant-First” and On-Demand Approach Based on Parenchymal Reduction

**DOI:** 10.1007/s00266-026-05954-5

**Published:** 2026-06-21

**Authors:** Salvatore Taglialatela Scafati, Gianmarco Polverino, Francesca Russo, Raffaele Russo, Francesco D’Andrea

**Affiliations:** 1 Plastiké STP, Plastic Surgery Clinic, Giugliano in Campania (Naples), Italy; 2 Unit of Plastic Surgery, University of Naples “Federico II”, Via Sergio Pansini, 5, 80131 Naples, Italy

**Keywords:** Augmentation mastopexy, Mammoplasty, Implant, Breast, Ptosis, Aesthetic surgery

## Abstract

Simultaneous augmentation mastopexy represents one of the most demanding procedures in aesthetic breast surgery, due to the need to balance volume enhancement with tissue tightening. Traditional techniques often struggle to maintain long-term stability, frequently resulting in recurrent ptosis, increased tension on the wound closure, and higher complication and revision rates. The Octopus Mastopexy was developed as a standardized refinement of the inverted-T approach, aiming to provide a reproducible, tension-free result while ensuring reliable nipple–areolar complex vascularization. A retrospective review was conducted of patients who underwent primary or secondary augmentation mastopexy using the Octopus technique between January 2019 and January 2024, all performed by the senior author. The technique is characterized by an implant-first sequence, selective and on-demand parenchymal reduction, and multivector pillar mobilization to evenly redistribute tension across the breast mound. Patient-reported satisfaction and quality-of-life outcomes were assessed using the BREAST-Q questionnaire, administered preoperatively and again at least 12 months postoperatively. Ninety-five patients were included, with a mean age of 39.2 years and a mean implant volume of 300 cc. No major complications occurred. Minor wound dehiscence was observed in two patients and managed conservatively, while six patients required revision for recurrent glandular laxity or weight change. BREAST-Q scores improved significantly across all measured domains, including satisfaction with breasts, physical well-being, and psychosocial well-being. These findings suggest that the Octopus Mastopexy provides a safe, stable, and reproducible solution for simultaneous breast augmentation and mastopexy, improving aesthetic outcomes while reducing tension-related complications and revision rates.

*Level of Evidence V* This journal requires that authors assign a level of evidence to each article. For a full description of these Evidence-Based Medicine ratings, please refer to the Table of Contents or the online Instructions to Authors www.springer.com/00266.

**Supplementary Information:**

The online version contains supplementary material available at (10.1007/s00266-026-05954-5).

## Introduction

Augmentation mastopexy remains one of the most frequently performed cosmetic surgical procedures worldwide, particularly among patients seeking to restore a youthful, elevated breast contour [1]. While several mastopexy techniques have been developed, ranging from circumareolar to vertical and inverted-T approaches, no single method has proven universally superior in addressing the complex anatomical and aesthetic challenges posed by moderate to severe ptosis, especially when combined with prosthetic augmentation [2]. Simultaneous augmentation mastopexy presents inherent challenges, as mastopexy requires tightening and reduction in both the skin envelope and glandular tissue, whereas augmentation increases volume and induces tissue expansion. These opposing biomechanical forces act simultaneously on the breast, potentially compromising tissue stability and increasing the risk of complications and revision surgery [3]. Thus, achieving a controlled balance between safety, projection, and long-term stability remains a key concern in breast surgery [4, 5].

We describe the “Octopus Mastopexy” (OM), a refinement of the inverted-T approach based on controlled parenchymal reduction. Applicable to augmentation mastopexy and implant exchange, it aims to enhance reproducibility, vascular safety, and long-term aesthetic stability.

## Materials and Methods

A retrospective analysis was performed on patients who underwent augmentation mastopexy using the OM technique between January 2019 and January 2024, performed by the first author. Patient-reported outcomes were evaluated using the BREAST-Q questionnaire preoperatively and at least 12 months postoperatively. Patients with BMI >30 kg/m^2^ or follow-up <12 months were excluded. Medical records were reviewed for surgical indications, technical details, and postoperative outcomes.

### Surgical Technique

**Fig. 1:**
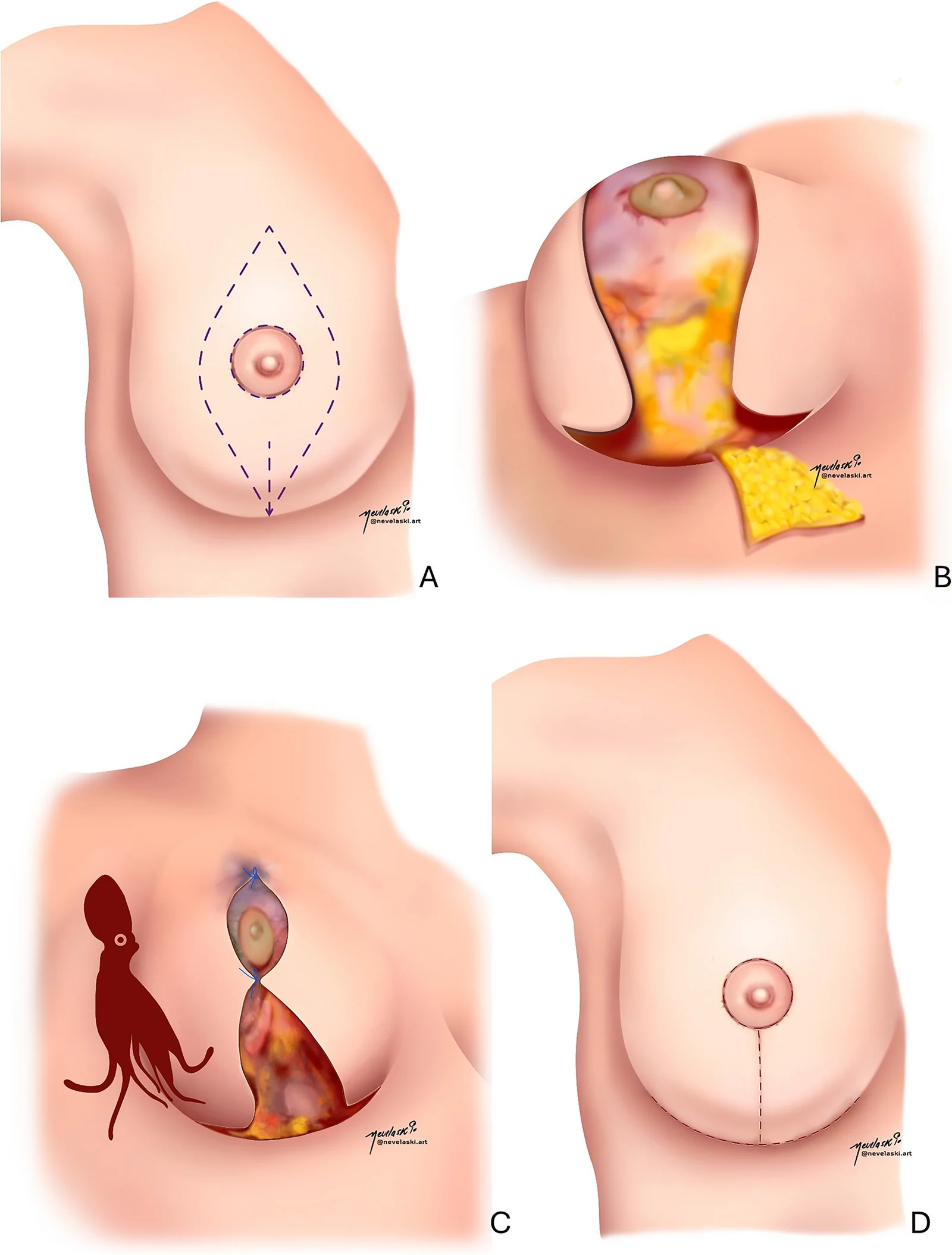
Schematic illustration of the Octopus Mastopexy technique (**A**–**D**), emphasizing the glandular reduction at the junction of the inferior quadrants (**B**) and the characteristic octopus-like morphology assumed by the mastopexy during one of its operative stages (**C**)

With the patient in the standing position, markings are made to define the new superior areolar border and the breast meridians. The inframammary fold is subsequently marked and adjusted according to the patient’s individual anatomy. The implant is inserted in either a subfascial or dual-plane pocket through an inframammary incision. In implant exchange cases, the same approach is used, performing capsulectomy or capsulotomy, followed by meticulous hemostasis. The inframammary fold is then anchored to the deep tissues, and the pocket is closed. Residual parenchymal laxity is assessed using the pinch test. From the predetermined upper areolar border, bilateral convex lines are traced toward the mid-IMF (Fig. 1A). Using a nipple marker, the periareolar and vertical incisions are made, followed by conservative de-epithelialization. *En bloc* glandular excision is performed at the junction of the lower quadrants, leaving a protective fascial layer over the implant (Fig. 1B). The periareolar dermis is incised medially and laterally, enabling controlled elevation of the nipple–areolar complex (NAC) while maintaining robust medial, superior, and lateral vascular supply. The NAC is gently elevated at the 12-o’clock position using a skin hook, with the incision configuration evoking the coordinated, fluid movement of an octopus in motion. With the NAC under traction, dissection of the lateral pillars is carried out using a Colorado needle along the plane of glandular excision. Breast pillars are subsequently mobilized and approximated with absorbable sutures while the areola is secured in its new superior position (Fig. 1C). After insetting the areola with cardinal sutures, the length of the future vertical scar is determined according to the type and dimensions of the selected implant. A horizontal excision of the fibroglandular tissue is eventually performed to create the horizontal component of the Wise pattern scar (Fig. 1D) (Video).

## Results

**Fig. 2:**
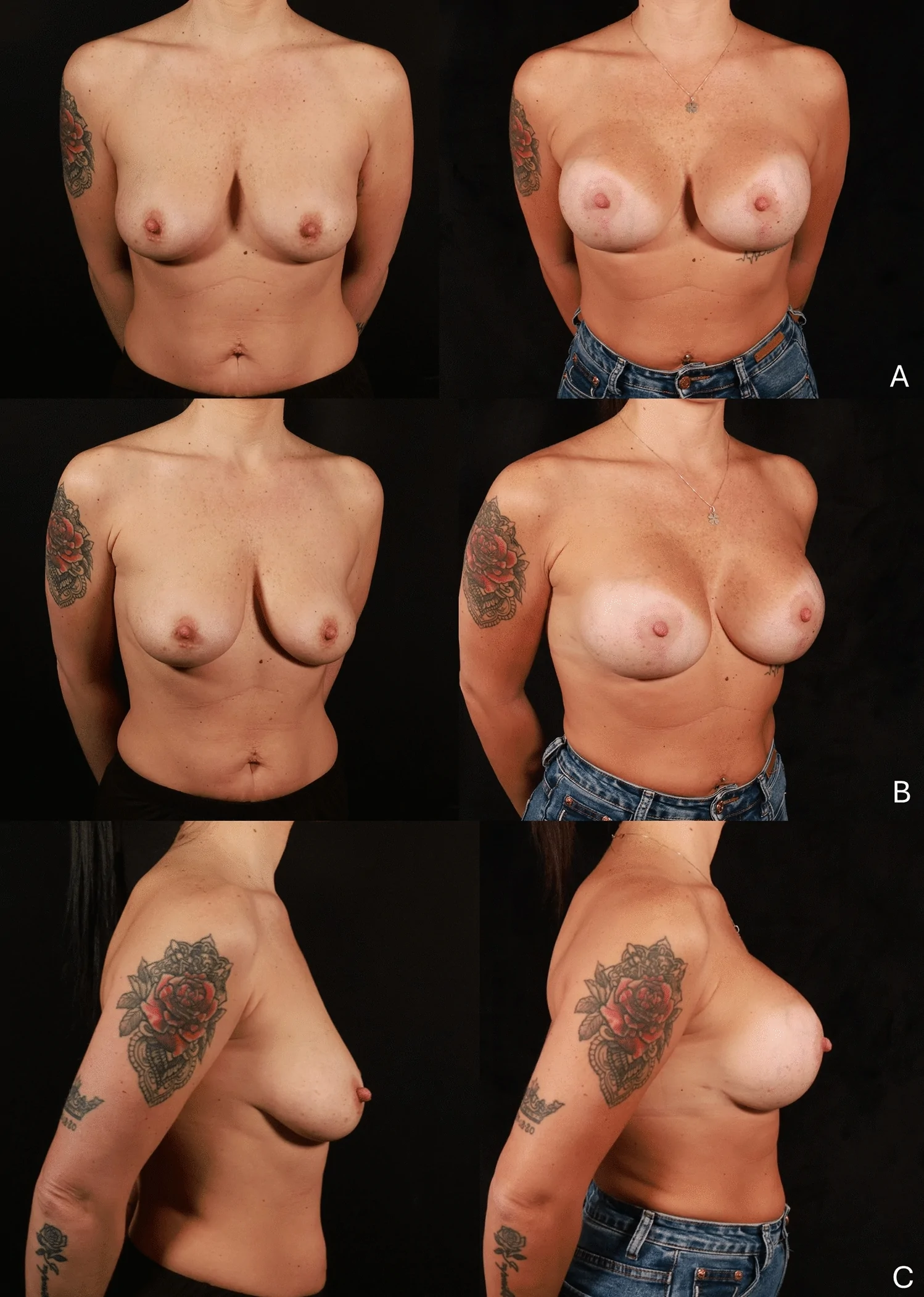
Preoperative (left) and 13-month postoperative (right) view of a 41-year-old patient following Octopus Mastopexy with 380-cc high-profile, round, polyurethane-coated implants. **A** Frontal view, **B** three quarter view, **C** lateral view

A total of 95 Caucasian patients underwent OM. In most cases, round polyurethane-coated implants were placed in a subfascial plane, whereas in 4 cases a dual-plane technique was performed using round silicone gel implants (Table 1). BREAST-Q scores improved significantly across all domains (*p* < 0.0001). At 12 months, 93.6% of patients reported satisfaction, confirming the procedure’s safety and lasting aesthetic results (Fig. 2).

**Table 1:** Demographics, complications, and BREAST-Q outcomes

Parameter	Preoperative (mean ± SD)	Postoperative (mean ± SD)	*P*-value (T-test)	Notes / Outcome
Number of patients	95	–	–	–
Age (years)	39.2 ± 8.1	–	–	–
Implant volume (cc)	300 (mean)	–	–	–
Major complications	None	–	–	Safety confirmed
Mastopexy revision	–	6 patients (6.3%),	–	Due to glandular laxity or weight fluctuation
Partial wound dehiscence	–	2 patients (2.1%)	–	Conservative management
Satisfaction with breasts (BREAST-Q)	43.87 ± 7.52	81.55 ± 6.41	< 0.0001	Significant improvement
Physical well-being (BREAST-Q)	42.78 ± 7.33	76.25 ± 8.43	< 0.0001	Significant improvement
Psychosocial well-being (BREAST-Q)	35.11 ± 7.29	71.13 ± 12.35	< 0.0001	Significant improvement
Overall satisfaction at 1 year	–	93.6% very satisfied	–	Stable results

## Discussion

Simultaneous augmentation mastopexy remains one of the most debated procedures in aesthetic breast surgery [6]. The intrinsic contradiction of increasing breast volume while simultaneously tightening the soft tissue envelope leads to higher complication and revision rates compared with augmentation alone [1–6]. A systematic review analyzing thousands of cases reported overall complication rates of approximately 13%, with recurrent ptosis around 5%, poor scarring near 4%, and revision surgery required in 10–17% of patients [7]. Large clinical series have similarly highlighted frequent challenges such as wound dehiscence and vascular compromise of the NAC [8, 9]. Despite the variety of pedicle designs, the current literature lacks consensus on how best to minimize tissue tension, calibrate skin resection, and preserve reliable NAC vascularization, representing a persistent gap in contemporary cosmetic breast surgery [8–11]. The OM technique addresses several of these limitations by providing a tailored parenchymal reduction without compromising NAC vascularization. The octopus-shaped incision pattern allows controlled, selective removal of glandular tissue from the lower quadrants. The key concept of the Octopus technique is to determine the exact amount of breast tissue to be removed after implant placement. By manually pinching the redundant tissue, marking it with a skin marker, and then performing the definitive excision, the surgeon is able to preserve the protective fascial layer overlying the implant. This approach allows for tension-free closure of the vertical breast pillars. Once the vertical component has been closed, excision of the excessive horizontal segment of the wise pattern is subsequently performed. In contrast, in traditional approaches, preoperative markings guide tissue excision, which is usually carried out before implant insertion. This may lead to significant difficulties during wound closure, as areas of excessive tension may develop due to the inability to accurately estimate the amount of redundant breast tissue. The practical rationale of the Octopus technique is based on selective reduction in the breast parenchyma, leading to a decrease in weight and redundancy of the lower pole, rather than relying on breast reshaping based solely on excision of the skin envelope. Furthermore, its reliance on multifocal vascular inflow ensures robust NAC perfusion while allowing greater areolar mobility. Another conceptual strength of this method lies in its implant-first strategy. By placing the implant before glandular tailoring, mastopexy is performed on demand, allowing direct and safe excision of redundant parenchymal tissue. This sequence minimizes the risk of inadequate volume distribution and recurrent ptosis while facilitating tension-free closure of the breast pillars, thereby optimizing scar quality and long-term stability. In addition, the stepwise mobilization of the glandular pillars provides structural reinforcement of the breast mound, enhancing projection and upper pole fullness while preventing overstretching of the soft tissues over the implant.

This on-demand mastopexy technique can be applied in cases of dual-plane or subfascial implant placement. In all cases in which the pinch test exceeds 1.5–2 cm, we prefer the use of polyurethane-coated implants, avoiding synthetic meshes. The latter would introduce additional foreign material, thereby increasing the risk of complications such as infection and seroma formation.

On the other hand, polyurethane-coated implants promote strong adhesion of the surrounding breast tissues through a “Velcro-like” effect [12], minimizing the risk of recurrent lower pole ptosis and progressive elongation over time. In cases of dual-plane placement with nano- or micro-textured implants, a synthetic mesh may be positioned through the initial inframammary fold incision. The superior edge of the mesh is sutured to the inferior border of the pectoralis major muscle, while its lower edge is fixed to the inframammary fold, thereby providing additional support to the implant and the lower pole of the breast. It should be specified that, in all patients included in this small series, no cases involving the use of synthetic meshes, commonly employed in our reconstructive practice, were included. Dual-plane implant placement without mesh was performed only when the pinch test was less than 1.5–2 cm, which occurred in only 4 out of 95 cases. The length of the vertical scar is determined only after closure of the vertical pillars and is primarily based on the projection of the implant, with the addition of approximately 1.5 cm. In cases involving polyurethane-coated implants, meticulous precision in defining the vertical length is essential, as this type of implant tends to maintain its position over time with minimal inferior settling. Conversely, when micro- or nano-textured implants are used, slightly shorter vertical limbs may be considered, given their greater tendency toward postoperative accommodation and lower pole relaxation.

The OM is a reproducible technique that combines on-demand inferior glandular tissue excision with implant-guided parenchymal tailoring and reliable NAC perfusion, while achieving a marked reduction in tension during breast mound closure.

OM provides a safe and anatomically reliable approach to simultaneous augmentation–mastopexy. Its implant-first, on-demand parenchymal tailoring and tension-free closure optimize vascular preservation, structural stability, and aesthetic outcomes, while probably reducing revision rates.

The technique described does not claim superiority over other mastopexy approaches; rather, it represents a rational evolution based on insights gained in recent years regarding the need to optimally manage the interaction between the intrinsic mechanical forces of the implant and the soft tissue envelope, particularly at the level of the lower breast pole.

The main limitations of this technical report include its single-surgeon design and retrospective nature, which carries an inherent risk of selection bias. Moreover, the relatively short follow-up period may not fully capture the long-term stability of outcomes or the true revision rates. Future studies comparing this approach with other techniques involving parenchymal reduction, ideally within a prospective randomized design, may be essential to validate long-term results.

## Supplementary Information

(Video S1_ESM MP4 93,541kb)
